# The crosstalk between SND1 and PDCD4 is associated with chemoresistance of non-small cell lung carcinoma cells

**DOI:** 10.1038/s41420-025-02310-5

**Published:** 2025-01-30

**Authors:** Yun Zhao, Shanel Dhani, Vladimir Gogvadze, Boris Zhivotovsky

**Affiliations:** 1 Department of Occupational and Environmental Health, School of Public Health, Medical College of Soochow University, Suzhou, China; 2 Institute of Environmental Medicine, Karolinska Institutet, Stockholm, Sweden; 3 Faculty of Medicine, MV Lomonosov Moscow State University, Moscow, Russia; 4 Engelhardt Institute of Molecular Biology, RAS, Moscow, Russia

**Keywords:** Lung cancer, Cell death

## Abstract

Lung cancer is the leading cause of cancer-related deaths worldwide. Non-small cell lung cancer (NSCLC) is highly resistant to chemo- or radiation therapy, which poses a huge challenge for treatment of advanced NSCLC. Previously, we demonstrated the oncogenic role of Tudor Staphylococcal nuclease (TSN, also known as Staphylococcal nuclease domain-containing protein 1, SND1), in regulating chemoresistance in NSCLC cells. Here, we showed that silencing of SND1 augmented the sensitivity of NSCLC cells to different chemotherapeutic drugs. Additionally, the expression of PDCD4 (a tumor suppressor highly associated with lung cancer) in NSCLC cells with low endogenous levels was attenuated by SND1 silencing, implying that SND1 might function as a molecular regulator upstream of PDCD4. PDCD4 is differentially expressed in various NSCLC cells. In the NSCLC cells (A549 and H23 cells) with low expression of PDCD4, despite the downregulation of PDCD4, silencing of SND1 still led to sensitization of NSCLC cells to treatment with different chemotherapeutic agents by the inhibition of autophagic activity. Thus, a novel correlation interlinking SND1 and PDCD4 in the regulation of NSCLC cells concerning chemotherapy was revealed, which contributes to understanding the mechanisms of chemoresistance in NSCLC.

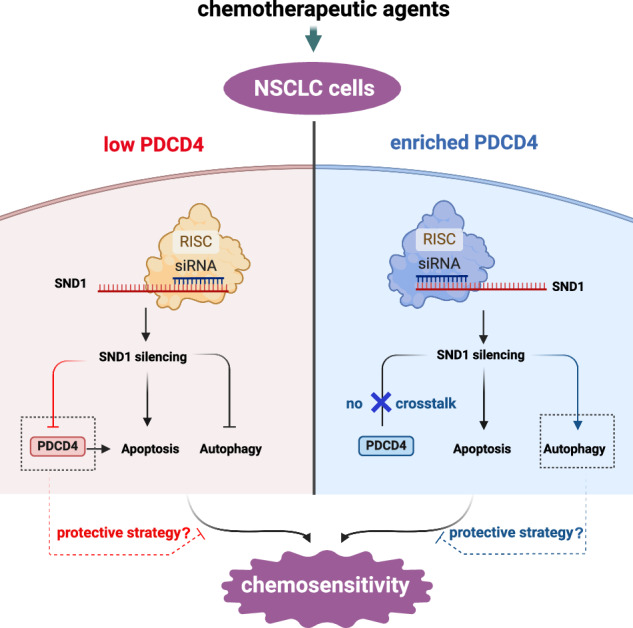

## Introduction

Lung cancer is the primary cause of cancer-related deaths around the world, accounting for the highest mortality among different cancer types [1]. The main histological types of lung cancer are small cell lung cancer (SCLC) and non-small cell lung cancer (NSCLC), of which lung adenocarcinoma (LUAD, approximately 60%) and lung squamous cell carcinoma (LUSC, approximately 30%) are the predominant subtypes with large cell carcinoma comprising about 10% of all NSCLC cases [2, 3]. Response to first-line treatment of lung cancer varies greatly between histological presentation. Compared to SCLC, NSCLC exhibits a relatively low sensitivity to chemo- or radiation therapy, therefore, the resistant cancer cells continue to grow and evade treatment, progressing to advanced stage where the more aggressive NSCLC remains largely incurable [4]. The resistance of cancer cells to programmed cell death (PCD) has been well attributed as a prerequisite for the development of cancer [5, 6], which suggests that improving our knowledge on potent PCD regulators that influence tumor resistance is crucial for enhancing the effectiveness of cancer therapy.

Various genes/proteins, e.g., p53 and BCL-2 family, are implicated in regulating apoptosis, the best-investigated form of PCD, upon chemotherapy, while the role of some apoptosis-associated genes/proteins playing in tumor cells for their poor response to chemotherapeutic agents is not yet well studied. Tudor staphylococcal nuclease (TSN, Tudor-SN, also known as Tudor domain-containing protein 11 (TDRD11), or Staphylococcal nuclease domain-containing protein 1, SND1, p100), is a multifunctional, evolutionarily conserved RNA-binding protein, involved in the regulation of gene expression of different processes ranging from transcription (as a transcriptional co-activator) to RNA interference (as a key component of RNA-induced silencing (RISC) complex) [7–9]. Additionally, SND1 is frequently overexpressed in multiple types of cancer, such as colon [10, 11], prostate [12], breast [13, 14], bladder [15] and hepatocellular carcinomas [16] and exhibits cytoprotective and oncogenic activity by promoting the proliferation of tumor cells [17–19]. Likewise, in our previous study, elevated SND1 expression and decreased chemoresistance of NSCLC cells to cisplatin was observed, and was reversed following the silencing of SND1 with an increase in apoptosis [20].

Earlier, we reported a series of genes with potential interaction with SND1 that may be involved in its proliferative role in NSCLC cells [20, 21]. Among the candidate genes identified, programmed cell death 4 (*PDCD4*, a gene whose product is capable of promoting apoptosis), as well as other genes closely associated with autophagy (e.g., *ATG10*) were found to be remarkably upregulated upon SND1 silencing in NSCLC cells. However, the functional relationship between PDCD4 and SND1 in the chemosensitivity of NSCLC cells induced by SND1 silencing are yet elusive. Therefore, potential molecular mechanisms underlying the modulation of lung cancer resistance conferred by SND1 need to be uncovered.

In the present study, we aimed to explore the crosstalk between SND1 and PDCD4 as a novel apoptosis-associated regulator in NSCLC development. Moreover, we intended to uncover how SND1 induces chemoresistance of NSCLC via modulation of apoptotic cell death and autophagy, with the intention of providing additional insight on the development of therapeutic targets to alleviate chemoresistance of NSCLC and improve the efficacy of lung adenocarcinoma treatments.

## Results

### Silencing of SND1 enhances the sensitivity of NSCLC cells to the treatment of chemotherapeutic drugs

**Fig. 1:**
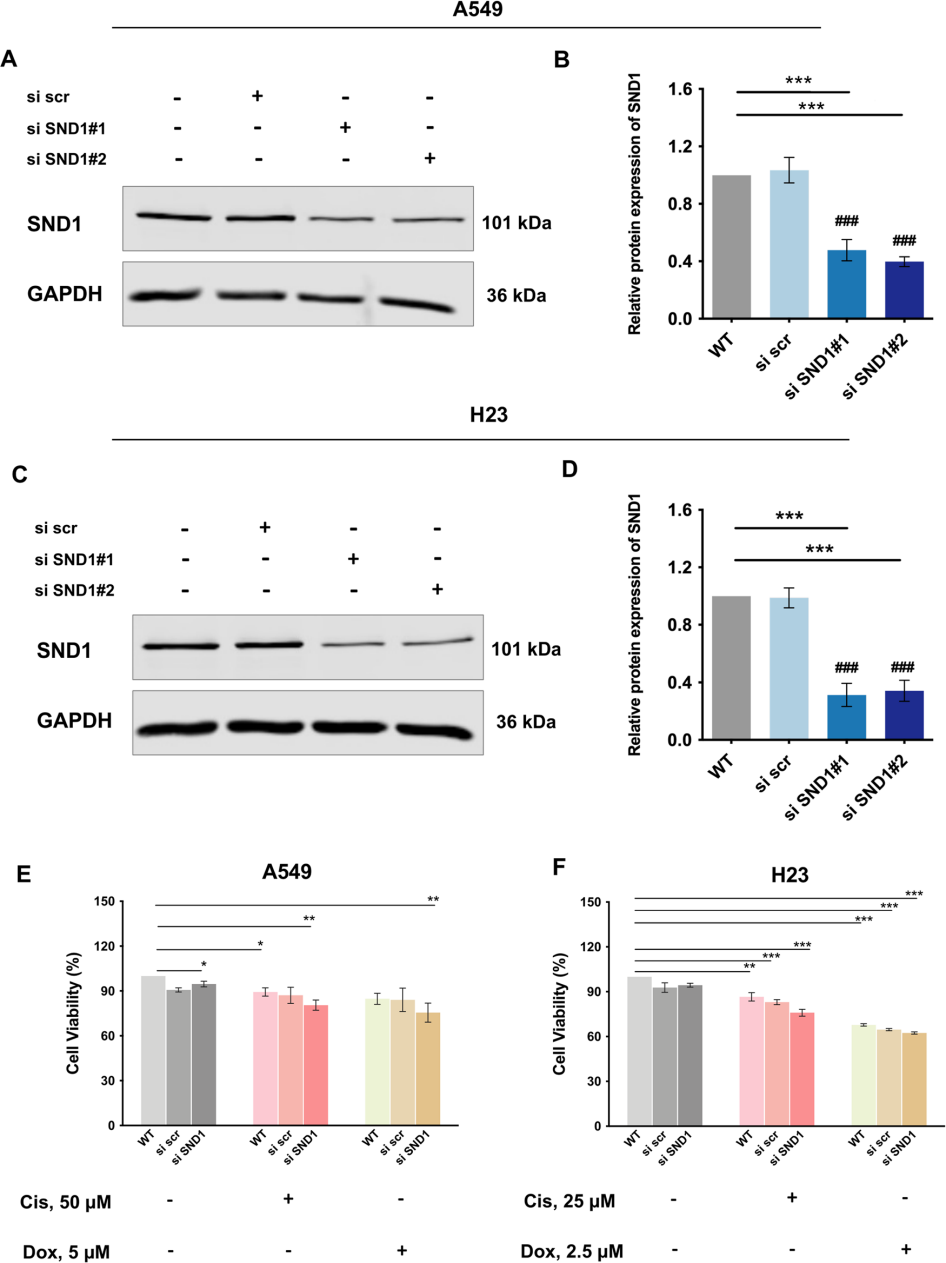
Silencing of SND1 increases the sensitivity of NSCLC cells to the treatment of chemotherapeutic chemicals. **A** The protein expression of SND1 in A549 cells upon silencing. **B** Densitometric analysis of the western blotting bands for SND1 in A549 cells normalized to GAPDH. ***p < 0.001, as compared to WT; ### *p* < 0.001, as compared to scramble siRNA (si scr). **C** The protein expression of SND1 in H23 cells upon silencing. **D** Densitometric analysis of the western blotting bands for SND1 in H23 cells normalized to GAPDH. ***p < 0.001, as compared to WT; ### *p* < 0.001, as compared to si scr. **E** Cell viability of A549 cells treated as indicated (cisplatin (Cis): 50 µM for 24 h; doxorubicin (Dox): 5 µM for 24 h). *p < 0.05, **p < 0.01, as compared to WT (no treatment). **F** Cell viability of H23 cells treated as indicated (Cis: 25 µM for 24 h; Dox: 2.5 µM for 24 h). **p < 0.01, ***p < 0.001, as compared to WT (no treatment).

In our previous study, SND1 silencing potentiated cisplatin-induced death of a cisplatin-resistant NSCLC cells A549 [20]. To validate these findings further, we first examined the chemosensitivity to several chemotherapeutic drugs including cisplatin, doxorubicin, oxaliplatin, 5-fluorouracil (5-FU), and etoposide in NSCLC cells, i.e., A549 (Fig. S1A–E) and H23 cells (Fig. S2A–E). For both NSCLC cell lines, an overall decrease in cell viability was observed with each chemotherapeutic agent in a dose-dependent manner. A549 cells (Fig. S1A, B) were less sensitive to cisplatin or doxorubicin compared to H23 cells (Fig. S2A, B). To confirm this, apoptotic cells were visualized by Hoechst staining following treatments with cisplatin (10 µM and 50 µM for A549; 5 µM and 25 µM for H23 cells) or doxorubicin (1 µM and 5 µM for A549; 1 µM and 2.5 µM for H23 cells) (Figs. S1F, 2F). A significant increase in apoptotic cells was observed in A549 cells treated with 50 µM cisplatin (*p* < 0.05) or 5 µM doxorubicin (*p* < 0.01) (Fig. S1F–f6), as well as in H23 cells treated with 25 µM cisplatin (*p* < 0.001) or 2.5 µM doxorubicin (*p* < 0.01) (Fig. S2F–f6), as expected. Subsequently, to understand how SND1 regulates the response of NSCLC cells to the chemotherapeutic agents, SND1 silencing was performed via the transfection with two different SND1-specific siRNAs in A549 cells (Fig. 1A, B) and H23 cells (Fig. 1C, D). Both cell lines were treated with cisplatin or doxorubicin for 24 h post-transfection at the doses chosen from the previous dose range tests (A549 cells: 50 µM cisplatin or 5 µM doxorubicin; H23 cells: 25 µM cisplatin or 2.5 µM doxorubicin). Compared to non-treated wild type (WT) cells, silencing of SND1 sensitized both A549 (cisplatin and doxorubicin: *p* < 0.01) (Fig. 1E) and H23 (cisplatin and doxorubicin: *p* < 0.001) cells (Fig. 1F) to both compounds. To further validate the chemosensitivity induced by SND1 silencing, A549 and H23 cells were treated with MG132 (10 µM), a proteasome inhibitor known to induce apoptosis in tumor cells, for 24 h. As expected, a significant decrease in cell viability was observed, demonstrating the oncogenic role of SND1 in NSCLC cells (Fig. S3A, B).

### PDCD4 overexpression stimulated apoptosis in NSCLC cells

Upon the elucidation of SND1-induced chemoresistance of NSCLC, a key question was subsequently put forward: do any potential molecular targets of SND1 contribute to the above-observed effect? Previously, we identified a series of SND1-regulated genes associated with the sensitization of NSCLC cells to cisplatin, of which, major functional targets closely involved in apoptotic cell death have been identified [20]. Among these apoptotic genes, *PDCD4*, a critical tumor suppressor gene that encodes a pro-apoptotic protein, which stimulates apoptosis via the inhibition of procaspase-3 mRNA translation [22], was identified to be upregulated by SND1 silencing at the gene level [20].

**Fig. 2:**
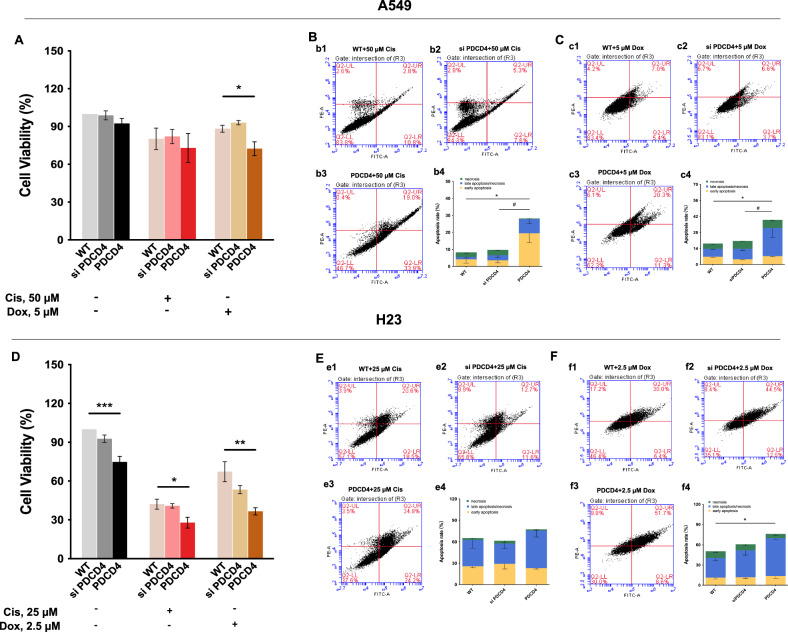
Overexpression of PDCD4 significantly increases the chemotherapeutic drugs induced apoptotic cell death. **A** Cell viability of A549 cells upon treatments with PDCD4 silencing or overexpression (Cis: 50 µM for 24 h; Dox: 5 µM for 24 h). **B** Flow cytometry analysis of apoptosis in A549 cells treated with 50 µM cisplatin for 24 h (b1: WT; b2: PDCD4 silencing; b3: PDCD4 overexpression; b4: the histogram of apoptosis ratio). **C** Flow cytometry analysis of apoptosis in A549 cells treated with 5 µM doxorubicin for 24 h (c1: WT; c2: PDCD4 silencing; c3: PDCD4 overexpression; c4: the histogram of apoptosis ratio). **D** Cell viability of H23 cells upon treatments with PDCD4 silencing or overexpression (Cis: 25 µM for 24 h; Dox: 2.5 µM for 24 h). **E** Flow cytometry analysis of apoptosis in H23 cells treated with 25 µM cisplatin for 24 h (e1: WT; e2: PDCD4 silencing; e3: PDCD4 overexpression; e4: the histogram of apoptosis ratio). **F** Flow cytometry analysis of apoptosis in H23 cells treated with 2.5 µM doxorubicin (f1: WT; f2: PDCD4 silencing; f3: PDCD4 overexpression; f4: the histogram of apoptosis ratio). *p < 0.05, **p < 0.01, ***p < 0.001, as compared to WT; #*p* < 0.05, as compared to si PDCD4.

To understand the role of PDCD4 in cell growth and apoptosis regulation, we first examined the effect of PDCD4 overexpression on viability and apoptosis in A549 cells (Fig. 2A–C) treated with cisplatin and doxorubicin. As expected, the overexpression of PDCD4 significantly sensitized A549 cells to treatment with doxorubicin (*p* < 0.05) but not with cisplatin, assessed by measurements of cell viability (Fig. 2A). Moreover, PDCD4 overexpression stimulated apoptosis in A549 cells treated with both agents (cisplatin: *p* < 0.05; doxorubicin: *p* < 0.05) (Fig. 2B, C). In H23 cells, characterized by low levels of PDCD4, overexpression of PDCD4 induced a significant reduction of cell viability with the treatment of both cisplatin (*p* < 0.05) and doxorubicin (*p* < 0.01) (Fig. 2D). Additionally, increased levels of apoptosis were observed in PDCD4-overexpressing H23 cells following treatment with cisplatin (slight increase with no significance, *p* > 0.05) (Fig. 2E) or doxorubicin (*p* < 0.05) (Fig. 2F). Thus, overexpression of PDCD4 significantly stimulates apoptotic cell death induced by antitumor drugs.

### SND1 functions as a molecular regulator upstream of PDCD4 in A549 and H23 cells

**Fig. 3:**
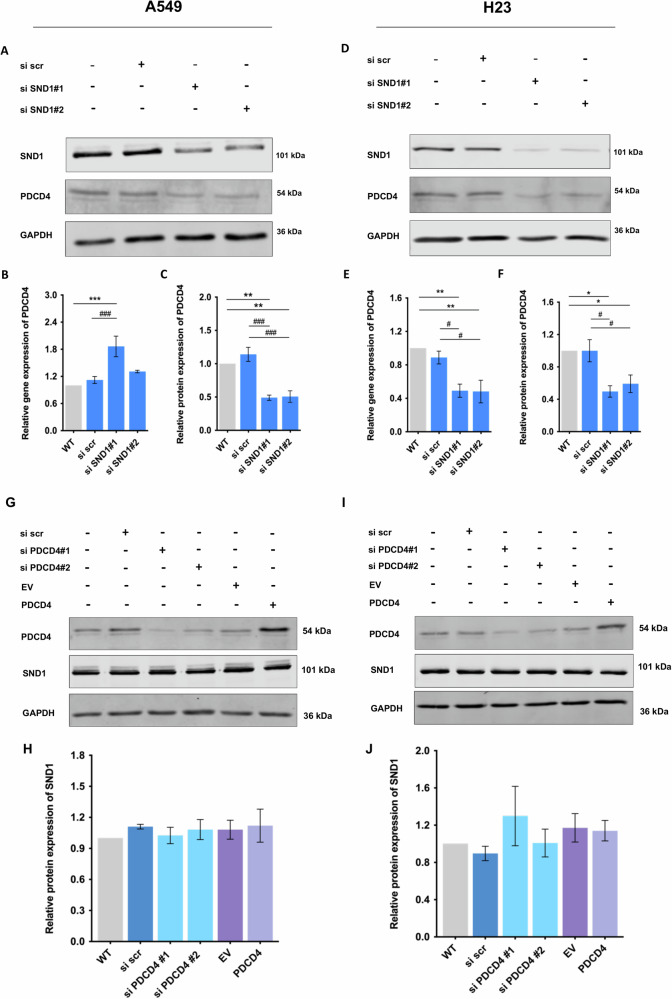
Silencing of SND1 downregulates PDCD4 as an upstream regulator. **A** The protein expression of PDCD4 in A549 cells upon SND1 silencing. **B** The relative gene exression of *PDCD4* in A549 cells upon SND1 silencing. **C** Densitometric analysis of the western blotting bands for PDCD4 in A549 cells normalized to GAPDH. **D** The protein expression of PDCD4 in H23 cells upon SND1 silencing. **E** The relative gene exression of *PDCD4* in H23 cells upon SND1 silencing. **F** Densitometric analysis of the western blotting bands for PDCD4 in H23 cells normalized to GAPDH. **G** The protein expression of SND1 in A549 cells upon PDCD4 silencing or overexpression. **H** Densitometric analysis of the western blotting bands for SND1 in A549 cells normalized to GAPDH. **I** The protein expression of SND1 in H23 cells upon PDCD4 silencing or overexpression. **J** Densitometric analysis of the western blotting bands for SND1 in H23 cells normalized to GAPDH. *p < 0.05, **p < 0.01, ***p < 0.001, as compared to WT; #*p* < 0.05, ###*p* < 0.001, as compared to si scr.

From our previous study, it was demonstrated that silencing of SND1 might result in the increased sensitivity of NSCLC to chemotherapeutic agents by promoting apoptosis via upregulating the level of PDCD4 [20]. However, the protein expression of PDCD4 was significantly downregulated upon SND1 silencing in A549 cells (*p* < 0.01) (Fig. 3A, C). On the other hand, gene expression was upregulated (si SND1#1: *p* < 0.001), which is consistent with our prior study (Fig. 3B). In addition, in H23 cells, the expression of PDCD4 decreased at both gene and protein levels upon the silencing of SND1 (Fig. 3D–F), while silencing or overexpression of PDCD4 had no effect on SND1 expression in either A549 (Fig. 3G, H) or H23 cells (Fig. 3I, J), suggesting that SND1 functioned as an upstream regulator of PDCD4. Furthermore, a positive correlation was revealed by the Spearman test between *PDCD4* and *SND1* expressions in normal lung tissues (LUAD normal: *p* = 0.0021, R = 0.4; LUSC normal: *p* = 0.0084, R = 0.37) (Fig. S4A, C) and lung tumors (LUAD: *p* = 0.0019, R = 0.14; LUSC: *p* = 0.0058, R = 0.13) (Fig. S4B, D), consistent with the protein expression trend of PDCD4 upon SND1 silencing observed above.

**Fig. 4:**
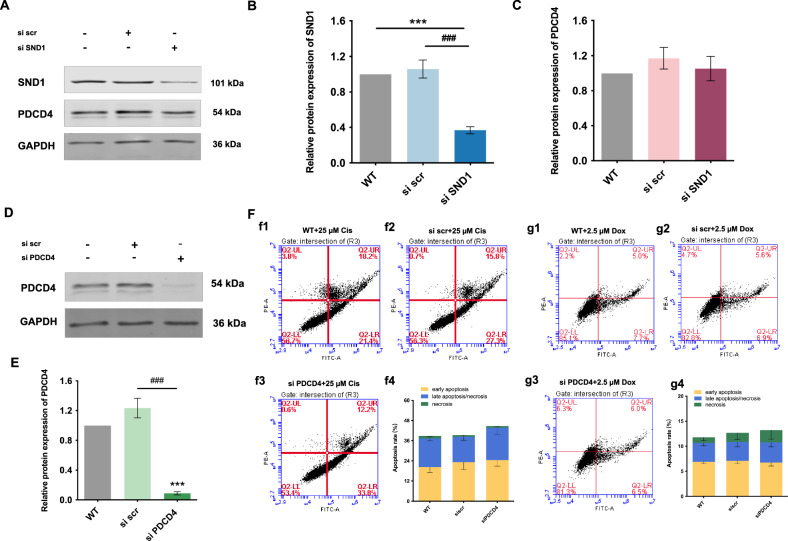
No crosstalk exists between SND1 and PDCD4 in H661 cells. **A** The protein expression of PDCD4 in H661 cells upon SND1 silencing. **B**, **C** Densitometric analysis of the western blotting bands for SND and PDCD4 in H661 cells normalized to GAPDH, respectively. **D**, **E** PDCD4 silencing in H661 cells. **F** Flow cytometry analysis of apoptosis in H661 cells treated with 25 µM cisplatin for 24 h (f1: WT; f2: scramble; f3: PDCD4 silencing; f4: the histogram of apoptosis ratio). **G** Flow cytometry analysis of apoptosis in H661 cells treated with 2.5 µM doxorubicin for 24 h (g1: WT; g2: scramble; g3: PDCD4 silencing; g4: the histogram of apoptosis ratio). ***p < 0.001, as compared to WT. ###*p* < 0.001, as compared to si scr.

**Fig. 5:**
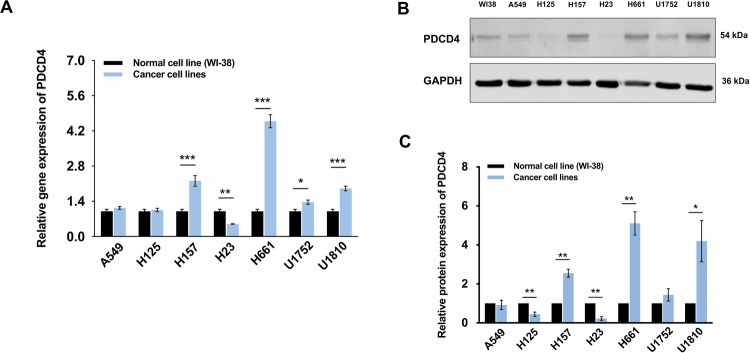
The gene and protein expressions of PDCD4 differ in normal human lung fibroblast cells and NSCLC cell lines. **A** The relative gene expression of *PDCD4* in NSCLC cells compared to that in normal lung cells. **B** The protein expression of PDCD4 in NSCLC cells compared to that in normal lung cells. **C** Densitometric analysis of the western blotting bands for PDCD4 normalized to GAPDH. *p < 0.05, **p < 0.01, ***p < 0.001, as compared to normal lung cell line (WI-38).

In contrast to observations in A549 or H23 cells, in H661 cells, SND1 silencing showed no effect on PDCD4 expression (Fig. 4A–C). Assessment on the viability of H661 cells revealed that both cisplatin (Fig. S5A) and doxorubicin (Fig. S5B) decreased cell viability in a dose-dependent manner. Silencing of PDCD4 (Fig. 4D, E) did not affect apoptosis in H661 cells treated with either of the drugs (Fig. 4F, G), implying that PDCD4, at least, did not play a key role in apoptosis regulation in H661 cells. Notably, *PDCD4* is differentially expressed in various NSCLC cells with significantly higher expression in several NSCLC cells, including H157 (*p* < 0.001), U1752 (*p* < 0.05), H661(*p* < 0.001), and U1810 (*p* < 0.001), compared to normal lung fibroblast cells, and markedly reduced levels in H23 cells (*p* < 0.01) (Fig. 5A). A higher level of PDCD4 protein was also observed in H157 (*p* < 0.01), H661 (*p* < 0.01) and U1810 cells (*p* < 0.05) (Fig. 5B, C). As aforementioned, some NSCLC cell lines revealed an increased level of PDCD4 despite its function as a general tumor suppressor, implying that PDCD4 might not always be a major player in regulating the apoptotic machinery in NSCLC.

### Silencing of SND1 differently affects autophagy in various NSCLC cells

**Fig. 6:**
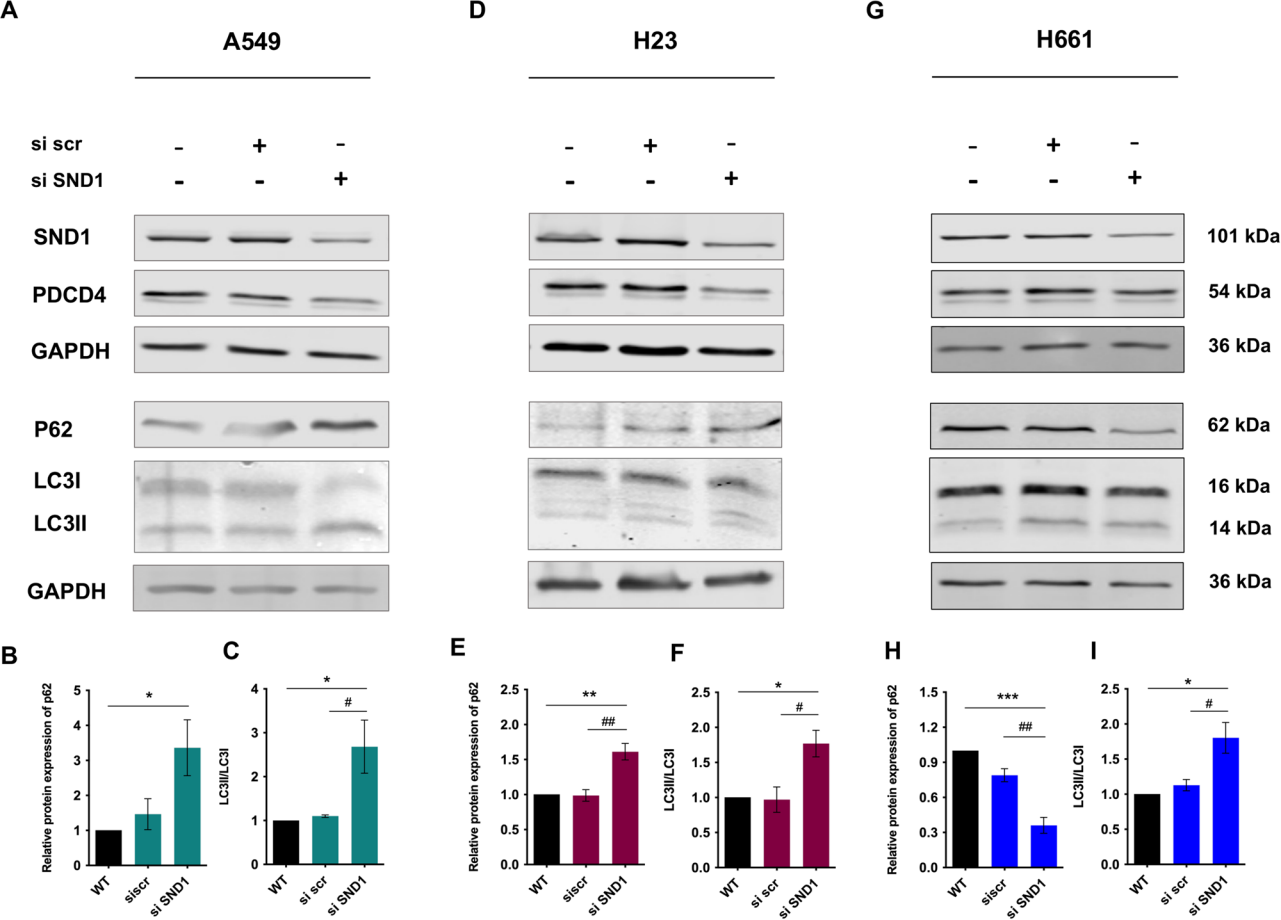
Silencing of SND1 differently affects autophagy in different NSCLC cells. The protein expressions of p62 and LC3 in A549 cells upon SND1 silencing (**A**: representative western blotting bands; **B**: relative protein expression of p62; **C**: LC3II/LC3I). The protein expression of p62 and LC3 in H23 cells upon SND1 silencing (**D**: representative western blotting bands; **E**: relative protein expression of p62; **F**: LC3II/LC3I). **G**–**I** The protein expression of p62 and LC3 in H661 cells upon SND1 silencing (**D**: representative western blotting bands; **E**: relative protein expression of p62; **F**: LC3II/LC3I). *p < 0.05, **p < 0.01, *** < 0.001, as compared to WT; # *p* < 0.05, ## *p* < 0.01, as compared to si scr.

Given that silencing of SND1 prompted the chemosensitivity of A549 and H23 cells, other cell death-related signaling or processes might be involved to combat the “plausibly protective” effect induced by down-expressed PDCD4. In our prior study, autophagy was shown to be inhibited by SND1 silencing in A549 cells. Consistent with these results, we observed autophagy inhibition with significant p62 accumulation (Fig. 6A, B) and LC3II formation from LC3I (Fig. 6A, C) in A549 cells. In addition, similar effects of autophagy suppression were revealed in H23 cells, evidenced by markedly high levels of p62 (Fig. 6D, E) and LC3II/LC3I (Fig. 6D, F). However, in contrast to A549 and H23 cells, p62 expression in H661 cells was significantly decreased (Fig. 6G, H) with elevated LC3I conversion to LC3II (Fig. 6G, I) following SND1 silencing, indicating the increased autophagic flux.

## Discussion

Chemotherapy is the standard of care for patients with NSCLC [23]. However, the development of resistance to chemotherapeutic drugs remains a substantial challenge in the treatment management for patients with this tumor type [24, 25]. Thus, unveiling the potential mechanisms of chemoresistance is of great importance for improving the efficacy of chemotherapeutic treatments for NSCLC.

SND1, a multifunctional protein and a key nuclease in several processes including RISC regulating transcription, mRNA splicing, and miRNA-mediated mRNA degradation, etc., is ubiquitously overexpressed in cancers where it generally functions as an oncogene [26]. SND1 was cleaved by caspase-3 during apoptosis, while uncleaved SND1 stimulated cell proliferation and protected cells from death [27]. Consistent with our previous study [20], in the current study, silencing SND1 rendered NSCLC cells more susceptible to the induction of apoptosis and induced sensitization of NSCLC cells to chemotherapeutic agents (Fig. 1E, F; Fig. S3A, B), demonstrating that SND1 functions as an essential mediator of NSCLC chemoresistance. Moreover, recent studies have also revealed that inhibition of SND1 promoted cisplatin-induced cell death in other cancer cells including bladder [15] and ovarian cancer cells [28], where the chemotherapy efficacy is generally low [29, 30], conferring the reversed chemoresistance. Taken together, the oncogenic potential of SND1 in cancer cells renders it to become a key player in mediating cell death.

Malfunctioning of apoptotic pathways represents one of the most common molecular changes that lead to chemoresistance [31], making the key molecules involved in regulating apoptosis important targets in cancer therapy. As shown in our previous study, the expression of several cell death-related genes was altered upon SND1 silencing, where PDCD4 was identified as a possible SND1-regulated candidate [20]. The role of PDCD4 in the translation of several tumor-suppressive genes involved in cancer cell proliferation, invasion, and metastasis is well-established [32]. Here, the overexpression of PDCD4 was shown to significantly induce apoptosis in A549 (Fig. 2B, C) and H23 cells (Fig. 2E, F), confirming the tumor-suppressive role of PDCD4. As demonstrated previously and herein, SND1 silencing in A549 cells stimulated *PDCD4* expression (Fig. 3B), implying that SND1 might be conferring chemoresistance in NSCLC cells via the suppression of PDCD4-mediated apoptotic cell death. However, contrary to the increased *PDCD4* expression, PDCD4 protein levels were significantly reduced in A549 cells (Fig. 3C) and H23 cells (Fig. 3F) upon SND1 silencing. We found that alterations of PDCD4 mRNA and protein level did not correlate in A549 cells (Fig. 3B, C). This result was consistent with findings by Kalinichenko et al., in which the PDCD4 protein-to-mRNA ratio was largely varied and was not directly correlated in human lung cancer cell lines [33]. The discrepancy between downregulated PDCD4 protein levels and upregulated gene expressions suggests the possibility of post-transcriptional regulation or protein degradation. One potential explanation is that SND1 silencing might influence pathways that destabilize PDCD4, which may be undergoing enhanced degradation via the ubiquitin-proteasome system. For instance, a previous research showed that pulsatile shear stress induces ubiquitin-proteasome–mediated degradation of PDCD4 in endothelial cells, supporting the involvement of this pathway in PDCD4 regulation [34]. Additionally, despite increased mRNA levels, efficiency of PDCD4 translation might be reduced, leading to lower protein expression. Furthermore, the upregulation of PDCD4 mRNA could be a compensatory feedback mechanism in response to decreased protein levels, as cells often attempt to restore protein homeostasis by increasing mRNA transcription when protein degradation is elevated [35, 36].

Furthermore, it remains unclear if the positive correlation between protein levels of SND1 and PDCD4 in NSCLC cells revealed in our current study was associated with the endogenous level of PDCD4. As a tumor suppressor, PDCD4 is frequently expressed at low levels in various tumor types including lung cancer [37–40]. At the same time, the level of PDCD4 in some NSCLC cell lines, e.g., H661 cells and U1810 cells, was remarkably higher than that in normal lung cells (Fig. 5A–C), which raised questions on the role between SND1 and PDCD4 in NSCLC cells with high PDCD4 levels. Surprisingly, we found that SND1 silencing played no regulatory role on PDCD4 in H661 cells (Fig. 4C), and that the silencing of PDCD4 in H661 cells did not affect apoptosis, implying that PDCD4 was potentially not a key player in SND1 silencing associated apoptosis in H661 cells. Taken together, the chemosensitivity caused by SND1 silencing in A549 and H23 cells, cells with low endogenous levels of PDCD4, did not result from PDCD4-mediated cell death. Conversely, the downregulation of PDCD4 might be a protective strategy for cells to resist the chemosensitivity induced by SND1 silencing.

Given that PDCD4 was downregulated upon silencing of SND1, alternative genes/pathways might be playing a leading role in silencing of SND1-induced cell death. TP53, a crucial tumor suppressor gene, inhibits the progression of cancers mainly by facilitating cell death such as apoptosis, ferroptosis, and autophagic cell death, in response to different cellular stresses [41]. Previous studies revealed the crosstalk between PDCD4 and p53, where PDCD4 was found to inhibit the translation of p53 mRNA and the phosphorylation of p53 [42, 43]. Additionally, a recent study suggested that p53 downregulated the protein expression of PDCD4 in H1299 cells, a type of NSCLC cell line derived from lymph nodes, implying that p53 might function as a novel regulator of PDCD4 [44]. In contrast to this study, we found no significant change of p53 protein expression upon SND1 silencing in both A549 (Fig. S6A, B) and H23 cells (Fig. S6C, D), suggesting other predominant pathways in the process of SND1 silencing-induced chemosensitivity.

Autophagy is an intracellular degradation process that occurs under cellular stress and promotes cell survival by breaking down and recycling organelles and proteins to maintain homeostasis [45–47]. Increasing evidence indicates that the inhibition of autophagy enhances the effectiveness of chemotherapy, thereby facilitating tumor reduction [48]. On the other hand, autophagy prevents tumorigenesis by suppressing the survival of cancer cells and inducing cell death [45]. Autophagy can serve as an effective escape mechanism for cancer, contributing to the development of resistance in various cancer types such as BRAF-mutated central nervous system tumors, melanoma, NSCLC, bladder cancer, and thyroid cancer [48]. Despite the dual role of autophagy in cancer, autophagy inhibition might be a reasonable and effective approach to reduce or reverse resistance to therapy in advanced cancer [49, 50]. In the present study, p62 accumulation and LC3 lipidation with a high level of LC3II/LC3I was observed upon SND1 silencing in A549 cells (Fig. 6B, C) and H23 cells (Fig. 6E, F) where levels of PDCD4 were significantly downregulated (Fig. 6A, D), suggesting the suppression of p62 degradation and the inhibition of autophagic flux. While SND1 silencing showed no effects on PDCD4 expression in H661 cells, autophagy was promoted as seen with decreased levels of p62 accumulation (Fig. 6G, H). and increased level of LC3II/I (Fig. 6G, I). Though knockdown of p62/SQSTM1 (sequestosome-1) led to the upregulation of PDCD4 in hepatoma cells [51], we found that p62 silencing had no regulatory effects on PDCD4 expression in NSCLC cells (Fig. S7A, B). Our finding further supports the suppression of autophagy as a key component in the increased chemosensitivity induced by SND1 silencing in some NSCLC cells with low levels of endogenous PDCD4. Interestingly, the suppression of autophagy was shown to inhibit the proliferation of NSCLC cells by cisplatin-induced caspase-dependent and -independent apoptosis [52].

As a novel tumor suppressor, PDCD4 interacts with eukaryotic translation initiation factor 4 A (eIF4A), crucially functioning as a translational repressor [53]. However, the mechanism underlying the tumor suppressive role of PDCD4, so far, is poorly understood. Even though we preliminarily revealed the potential crosstalk between PDCD4 and SND1 in various NSCLC cells, the in-depth mechanism of how SND1 regulates PDCD4 is still vague. It is unclear, why PDCD4 is substantially downregulated in some types of NSCLC cells (A549 and H23 cells) after SND1 silencing, which is proved to induce chemosensitivity. The assumption raised in the current study is that these NSCLC cells might be inclined to decrease the level of PDCD4 to partially resist the chemosensitivity induced by SND1 deficiency via inhibiting the autophagy level, mainly due to the low endogenous expression of PDCD4 in such cells. In this regard, other alternative or predominant tumor suppressors/apoptotic modulators may play a bigger role in SND1 silencing-induced chemosensitivity in NSCLC cells. Moreover, the fact that some lung cancer cells are characterized by high expression of PDCD4 suggests that this protein is not always tumor suppressive. The gray area between SND1 and PDCD4, in the context of chemotherapy on NSCLC, requires further investigation.

## Conclusions

In conclusion, we revealed the oncogenic function of SND1, whose deficiency is closely associated with the augmented chemosensitivity of NSCLC cells. Particularly, this study demonstrated the involvement of autophagy as a key factor in response to SND1 silencing in NSCLC cells and, moreover, a novel correlation interlinking SND1 and PDCD4 in the regulation of NSCLC cells concerning chemotherapy. Thus, our results contribute to understanding the mechanisms of chemoresistance in NSCLC.

## Materials and Methods

### Cell lines

Normal human lung fibroblast cell line WI-38 (AG06814) (purchased from ATCC) as well as human NSCLC cell lines, i.e., A549, H23, H661, H125, H157, U1752 and U1810, were used in this study. All NSCLC cells were cultured in Roswell Park Memorial Institute-1640 (RPMI-1640) medium, while WI-38 cells were cultured in Dulbecco’s Modified Eagle Medium (DMEM). Each medium type was supplemented with 10% (V/V) heat-inactivated fetal bovine serum (FBS), 2 mM L-glutamine, and 1% penicillin-streptomycin (100 μg/mL). Cells were cultured in a humidified incubator with 5% CO_2_ at 37 °C.

### Transfection

All the siRNAs used in this study, i.e., scramble siRNAs, SND1-specific siRNAs, PDCD4-specific siRNAs, and p62-specific siRNAs, were purchased from Invitrogen (ThermoFisher Scientific, Carlsbad, CA, USA). The pcDNA3.1-PDCD4 plasmid was generously gifted by Dr. Hsin-Sheng Yang (University of Kentucky, KY, USA). Briefly, cells were seeded to reach 70–90% confluency at the time of transfection. The transfection with siRNAs and plasmids was performed using Lipofectamine 3000 transfection reagent (L3000008, Invitrogen) according to the manufacturer’s protocol.

### Chemotherapeutic drugs treatment and cytotoxicity assay

Cytotoxicity tests were conducted using CellTiter 96® non-radioactive cell proliferation assay (Promega, Madison, WI) according to the manufacturer’s protocol. Briefly, 80 µL cells were seeded into 96-well cell culture plates at a density of 1.25 × 10^5^ cells/mL (1 × 10^4^ cells/well) one day prior to treatment. Cells were treated with cisplatin, doxorubicin, oxaliplatin, 5-FU and etoposide, respectively, for 24 or 48 h within different dose ranges as indicated in the Supplementary Information. All the chemotherapeutic drugs were purchased from Sigma-Aldrich (St. Louis, MO, USA). At the end of treatment, 15 µL of dye solution was added into each well and incubated at 37 °C in a humidified, 5% CO_2_ atmosphere. After 4 h of incubation, solubilization solution (100 µL/well) was gently added to stop the reactions. The plate was incubated for 1 h at 37 °C and the absorbance was recorded using a 96-well plate reader at 570 nm. Based on the cell viability tests, the doses of chemotherapeutic drugs used for the post-transfection treatments are as follows: 50 µM cisplatin and 5 µM doxorubicin were used to treat A549 cells for 24 h, while 25 µM cisplatin and 2.5 µM doxorubicin were used to treat H23 and H661 cells. H23 cells were more sensitive to doxorubicin treatment upon transfection, thus, the media containing Lipofectamine 3000 was removed after 6-hour transfection and supplemented with fresh media.

### Western blotting

Cells were seeded in 6-well plates at a density of 3 × 10^5^ cells/well. Total proteins were extracted using RIPA lysis buffer (ThermoFisher Scientific, Carlsbad, CA, USA) supplemented with an EDTA-free protease inhibitor cocktail (Sigma-Aldrich, St. Louis, MO, USA). The cell lysates were collected for centrifugation (12,000 rpm, 4 °C, 20 min) to isolate total proteins in the supernatant. BCA assay was performed to quantify the protein concentrations. Primary antibodies against the target proteins were as follows: anti-SND1 (ab65078) from Abcam (Cambridge, UK), anti-PDCD4 (#9535), anti-p53 (#9282), anti-p62 (#5114), and anti-LC3 A/B (#12741), all from Cell Signaling Technology (MA, USA); anti-GAPDH (MA5-35235, used as the reference protein) from Invitrogen (ThermoFisher Scientific, Carlsbad, CA, USA). Fluorescent anti-rabbit and anti-mouse secondary antibodies from LI-COR Biosicences (Lincoln, NE, USA) were used. Proteins’ densitometry was analyzed by ImageStudio software. The blots of different proteins shown in the figures represent one out of three independent experiments. All the original uncropped western blots are seen in Supplementary Information.

### Real-time (RT)-Quantitative (q) PCR

Expression of *PDCD4* gene was measured by RT-qPCR. Briefly, total RNA was extracted using the Trizol reagent from Sigma-Aldrich (St. Louis, MO, USA). The isolated RNA samples were reverse transcribed into complementary DNA (cDNA) using a High-Capacity cDNA reverse transcription kit from Applied Biosystems (ThermoFisher Scientific, Carlsbad, CA, USA). SYBR Green Master Mix was used to perform qPCR, which was run on Applied Biosystems™ 7500 RT-PCR system (ThermoFisher Scientific, Carlsbad, CA, USA). *β-Actin* was used as the reference gene. The primer sequences for *PDCD4*, and *β-Actin* are listed in the Supplementary Information (Table S1).

### Annexin V and propidium iodide (PI) staining

The detection and quantification of apoptosis in A549, H23, and H661 cells upon treatment with chemotherapeutic drugs was conducted using an Annexin-V-FLUOS staining kit (Roche, Basel, Switzerland). Briefly, cells were seeded into a 12-well plate at a density of 1 × 10^5^ cells/well for transfection and subsequent drug treatments. After treatment, the cells were collected and stained for Annexin V and PI as per the manufacturer’s instructions. A total of 1 × 10^6^ cells per sample were analyzed with a BD Accuri™ C6 Plus flow cytometer (BD Biosciences, NJ, USA).

### Hoechst staining and confocal microscopy

A549 and H23 cells were seeded in 24-well cell culture plates at the densities of 5 × 10^4^ cell/well for A549 cells and 1 × 10^5^ cell/well for H23 cells, respectively. A549 cells were treated with cisplatin and doxorubicin and stained with Hoechst 33342 at a concentration of 10 μg/mL. The stained cells were examined using a Zeiss LSM880 confocal microscope (Carl Zeiss MicroImaging, Göttingen, Germany).

### Statistical analysis

The data are represented as the Mean ± SEM. The results were presented as three independent experiments. SPSS was used to perform statistical analysis. One-way ANOVA followed by an LSD test was applied for the analysis of data with equal variance, while the Dunnet T3 post hoc test was applied with unequal variance. An unpaired *t*-test was used to analyze gene and protein expressions of PDCD4 in WI-38 cells and multiple NSCLC cells. For the flow cytometry, the mean of each specific quadrant from three biological replicates was calculated. The Spearman test was performed on GEPIA2 [54] to analyze the correlation between *SND1* and *PDCD4*. *P* < 0.05 was considered statistically significant.

## Supplementary information

Supplementary Material

Original blots

## Data Availability

Original data are available upon request.
