# Supplementary material for: The crosstalk between SND1 and PDCD4 is associated with chemoresistance of non-small cell lung carcinoma cells
Source: Cell Death Discov. 2025 Jan 30;11:34. doi: 10.1038/s41420-025-02310-5 (PMC11782486; doi:10.1038/s41420-025-02310-5)
Supplement: Supplementary file 1 — Supplementary Material [file 41420_2025_2310_MOESM1_ESM.pdf]

.Supplementary Information

The Crosstalk Between SND1 and PDCD4 Is Associated with Chemoresistance of Non-Small Cell Lung Carcinoma Cells

Yun Zhao[1,2], Shanel Dhani[2], Vladimir Gogvadze[2,3], Boris Zhivotovsky[2,3,4] *

[1] Department of Occupational and Environmental Health, School of Public Health, Suzhou Medical College of Soochow University, Suzhou, China.

[2] Institute of Environmental Medicine, Karolinska Institutet, Box 210, 17177 Stockholm, Sweden.

[3] Faculty of Medicine, MV Lomonosov Moscow State University, 119991 Moscow, Russia

[4] Engelhardt Institute of Molecular Biology, RAS, 119991 Moscow, Russia

* Corresponding Author:

Boris Zhivotovsky: boris.zhivotovsky@ki.se

Table S1. Primer sequences used in RT-qPCR analysis

Genes	Primer sequences (5'-3')
PDCD4	F: GTTGGCAGTATCCTTAGCATTGG<br>R: TCCACATCAGTTGTGCTCATTAC
β-Actin	F: GCTGTGCTATCCCTGTACGC<br>R: GAGGGCATACCCCTCGTAGA

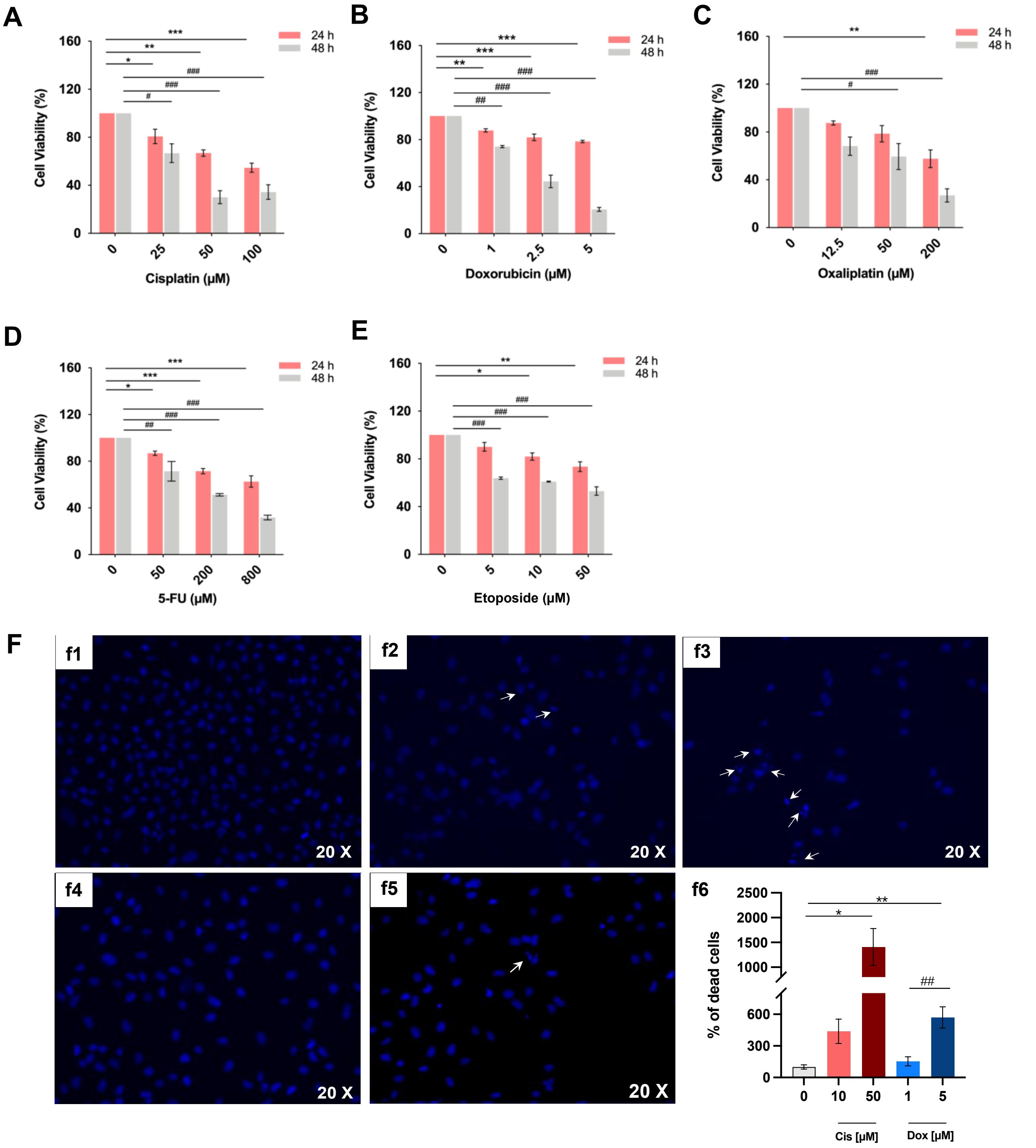

Figure S1. Cell viability and apoptosis induced by different chemotherapeutic drugs in A549 cells. A-E. Cell viability of A549 cells upon chemotherapeutic drugs' treatments for 24 hours and 48 hours, respectively. * $p < 0.05$; ** $p < 0.01$, *** $p < 0.001$, as compared to control (no treatment, 24 hours); # $p < 0.05$, ## $p < 0.01$, ### $p < 0.001$, as compared to control (no treatment, 48 hours). **F**. Hoechst staining for cell apoptosis upon different treatments (20 X) (f1: control with no treatment; f2: 10 μM Cis; f3: 50 μM Cis; f4: 1 μM Dox; f5: 5 μM Dox; f6, quantification of Hoechst staining. * $p < 0.05$; ** $p < 0.01$, as compared to control; ## $p < 0.01$, as compared to 1 μM Dox).

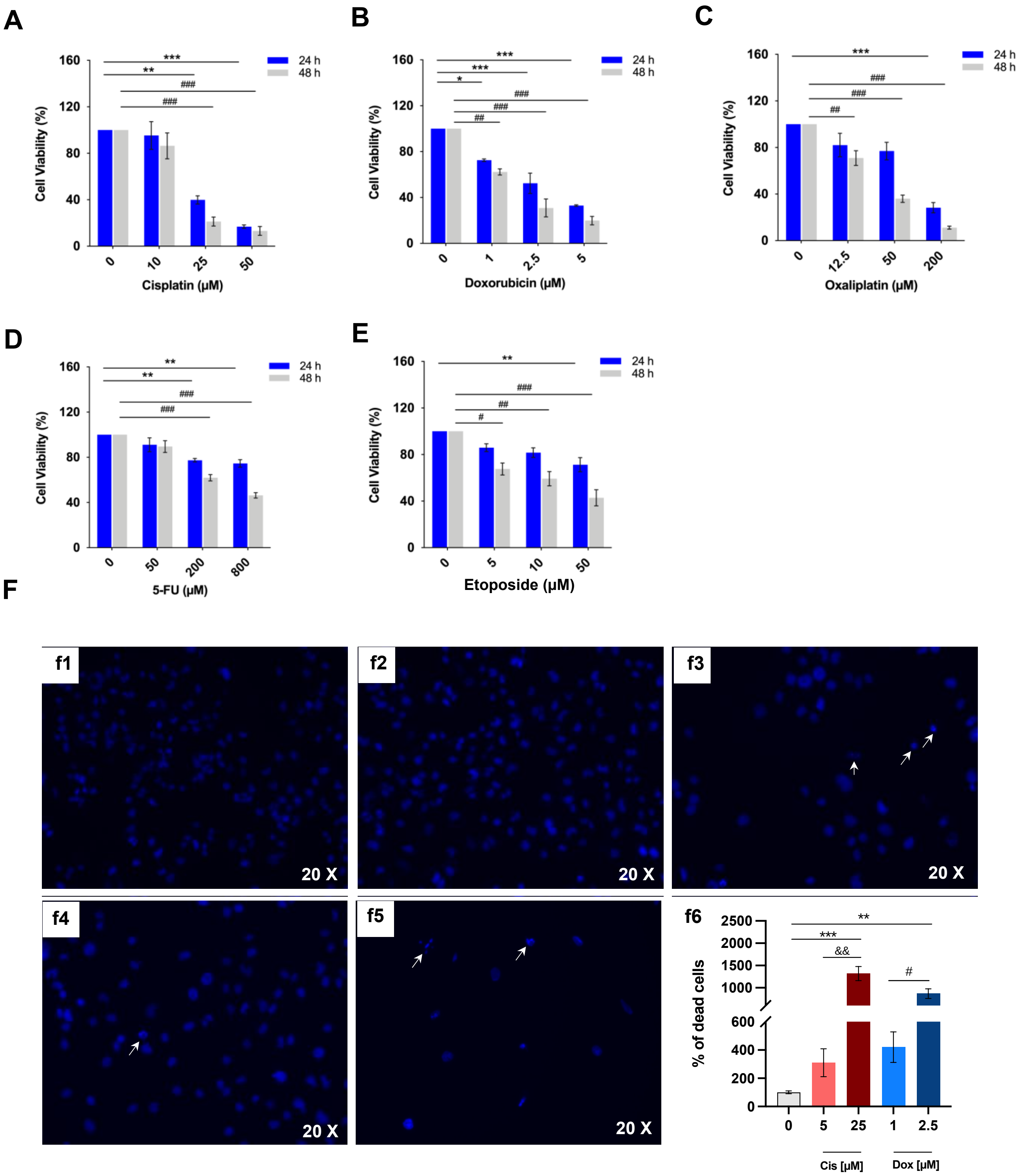

Figure S2. Cell viability and apoptosis induced by different chemotherapeutic drugs in H23 cells. A-E. Cell viability of H23 cells upon chemotherapeutic drugs' treatments for 24 hours and 48 hours, respectively. * $p < 0.05$, ** $p < 0.01$, *** $p < 0.001$, as compared to control (no treatment, 24 hours); # $p < 0.05$, ## $p < 0.01$, ### $p < 0.001$, as compared to control (no treatment, 48 hours). **F**. Hoechst staining for cell apoptosis upon different treatments (20 X) (f1: control with no treatment; f2: 5 μM Cis; f3: 25 μM Cis; f4: 1 μM Dox; f5: 2.5 μM Dox; f6: quantification of Hoechst staining. ** $p < 0.01$, *** $p < 0.001$, as compared to control; && $p < 0.01$, as compared to 5 μM Cis; # $p < 0.05$, as compared to 1 μM Dox).

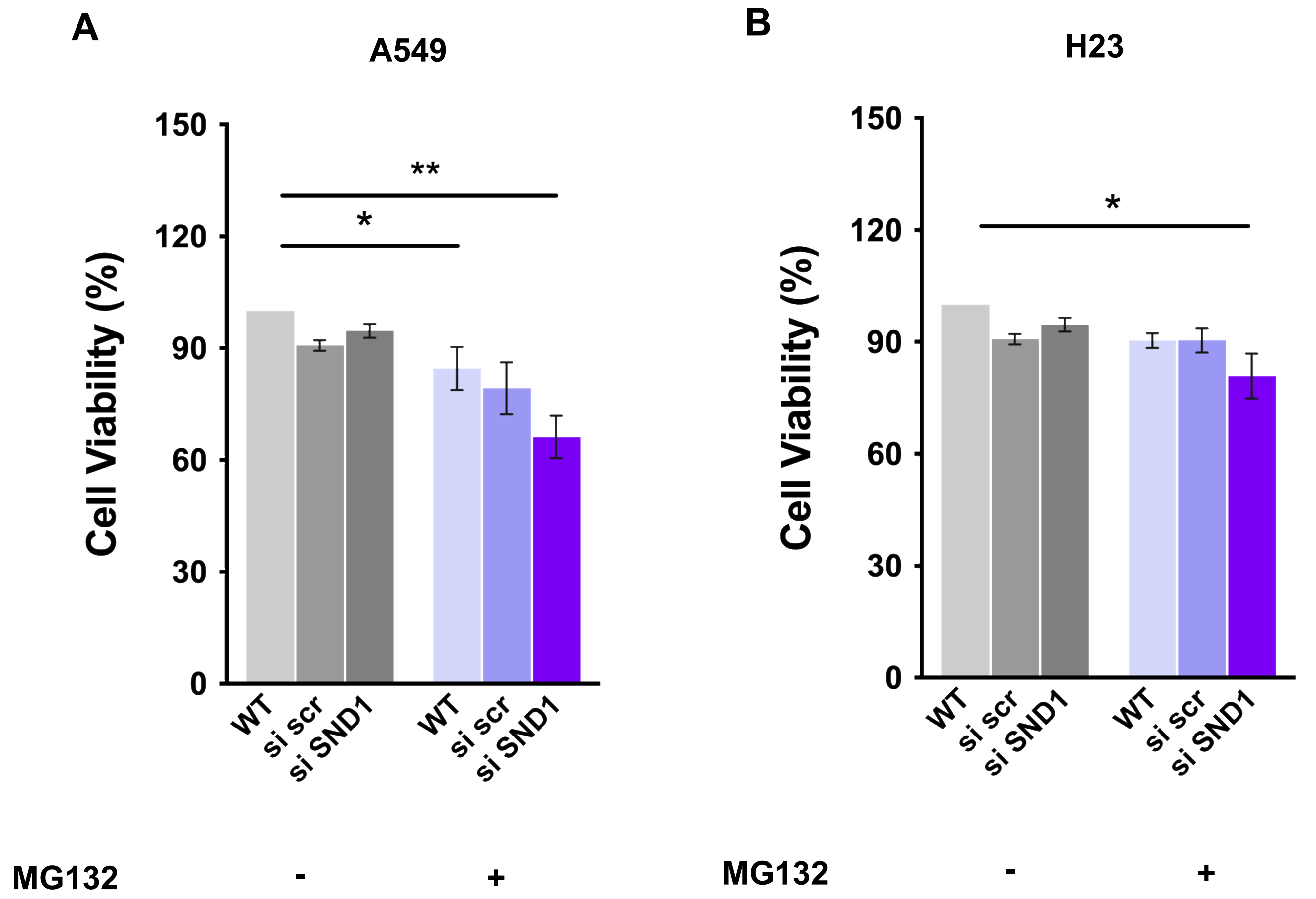

Figure S3. Silencing of SND1 increases the sensitivity of NSCLC cells to MG132, a proteasome inhibitor inducing apoptosis. A. Cell viality of A549 cells upon MG132 treatment with SND1 silencing (MG132: 10 μM for 24 hours). **B**. Cell viality of H23 cells upon MG132 treatment with SND1 silencing (MG132: 10 μM for 24 hours). * $p < 0.05$, ** $p < 0.01$, as compared to WT.

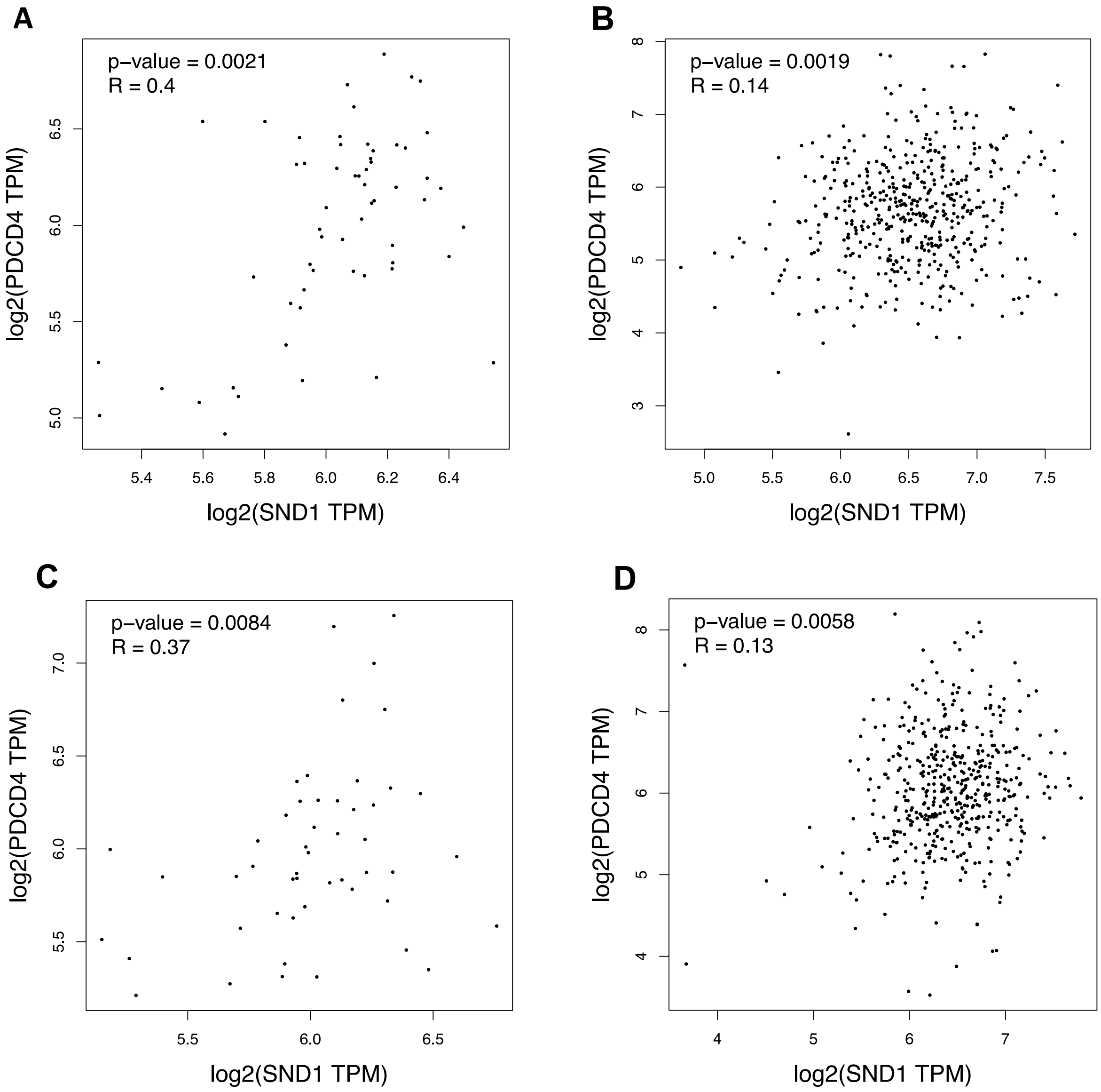

Figure S4. Spearman correlation analysis of *PDCD4* and *SND1* in TCGA. A. TCGA_LUAD normal. **B**. TCGA_LUAD. **C**. TCGA_LUSC normal. **D**. TCGA_LUSC.

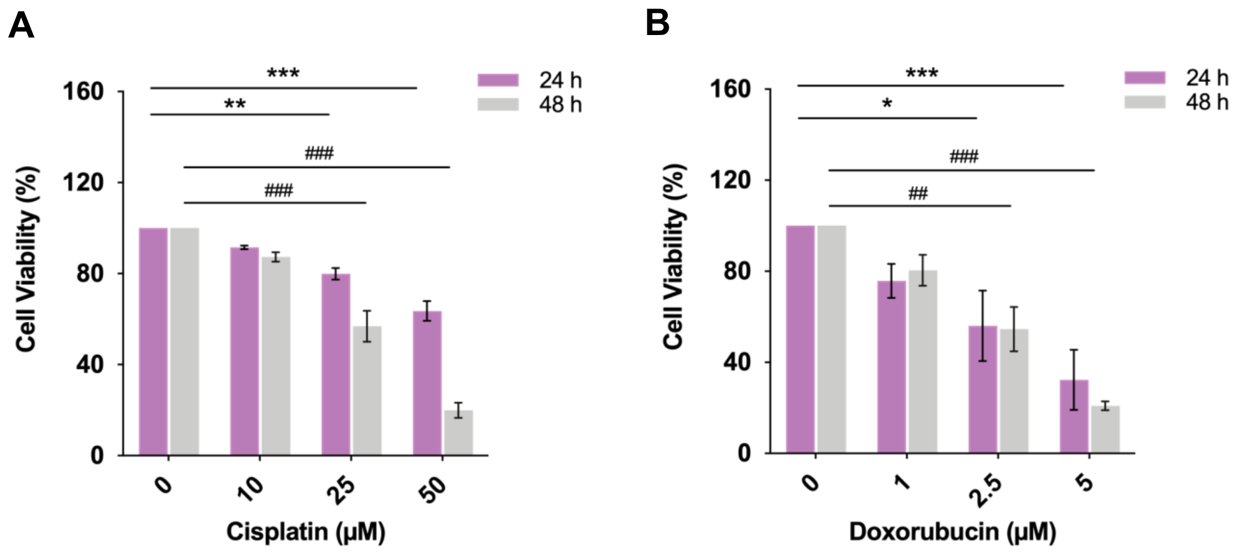

Figure S5. Cell viability induced by different chemotherapeutic drugs in H661 cells. A. Cell viability of H661 cells upon cisplatin treatment for 24 hours and 48 hours, respectively. **B**. Cell viability of H661 cells upon doxorubicin treatment for 24 hours and 48 hours, respectively. * $p < 0.05$; ** $p < 0.01$, *** $p < 0.001$, as compared to control (no treatment, 24 hours); ## $p < 0.01$, ### $p < 0.001$, as compared to control (no treatment, 48 hours).

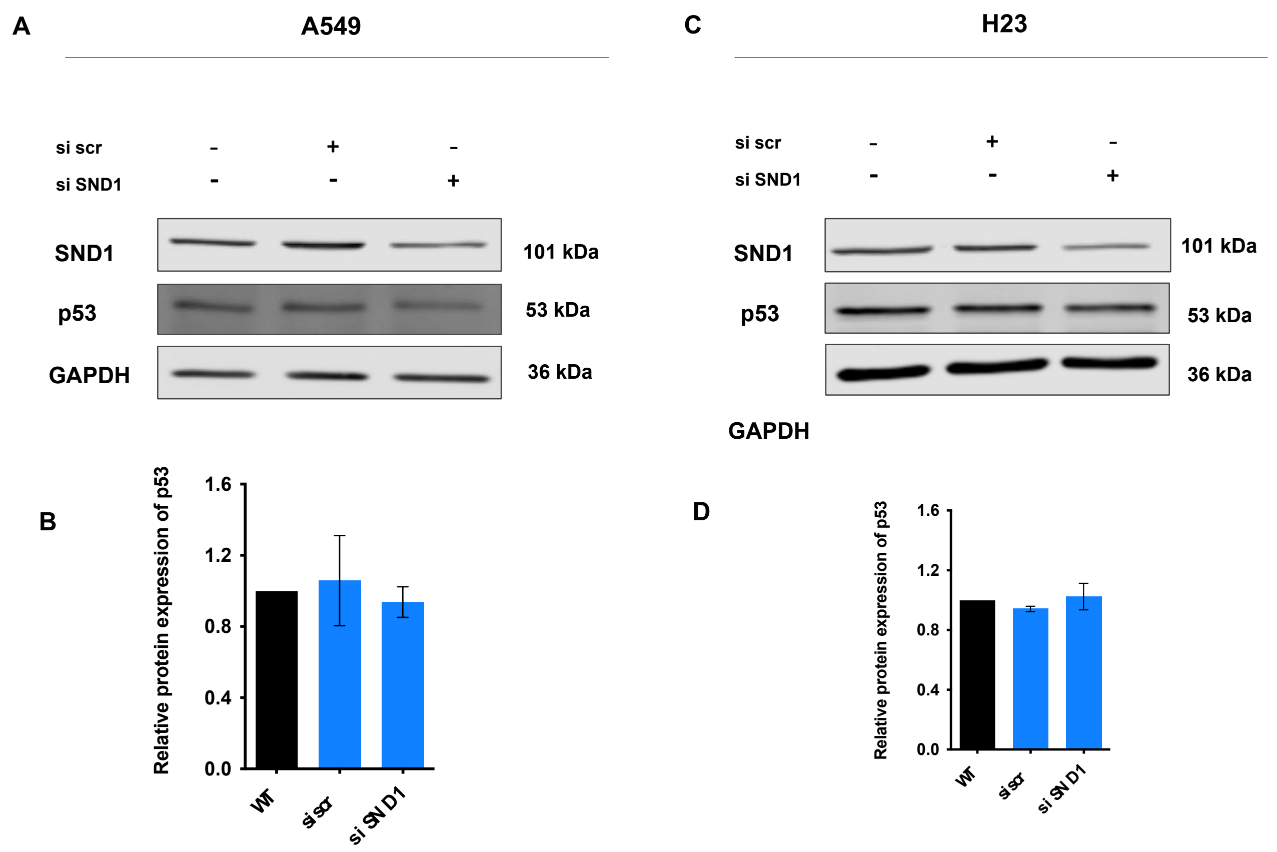

Figure S6. Silencing of SND1 differently affects other tumor suppressors in different NSCLC cells. A and B. The protein expression of p53 in A549 cells upon SND1 silencing (A: representative western blotting bands; B: relative protein expression of p53). **C and D.** The protein expression of p53 in H23 cells upon SND1 silencing (C: representative western blotting bands; D: relative protein expression of p53).

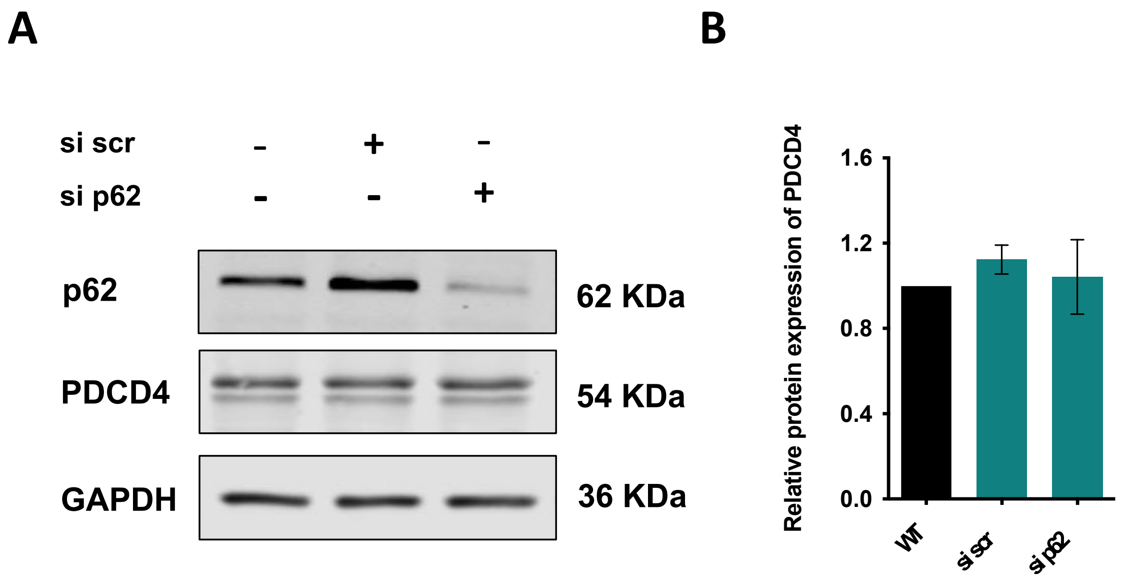

Figure S7. Silencing of p62 has no influence on PDCD4 expression in A549 cells. A and B. The relative expression of PDCD4 in A549 cells upon p62 silencing. (A: representative western blotting bands; B: relative protein expression of PDCD4)
