# Supplementary material for: The crosstalk between SND1 and PDCD4 is associated with chemoresistance of non-small cell lung carcinoma cells
Source: Cell Death Discov. 2025 Jan 30;11:34. doi: 10.1038/s41420-025-02310-5 (PMC11782486; doi:10.1038/s41420-025-02310-5)
Supplement: Supplementary file 2 — Original blots [file 41420_2025_2310_MOESM2_ESM.pdf]

Supplementary Information_original western blots

The Crosstalk Between SND1 and PDCD4 Is Associated with Chemoresistance of Non-Small Cell Lung Carcinoma Cells

Yun Zhao[1,2], Shanel Dhani[2], Vladimir Gogvadze[2,3], Boris Zhivotovsky[2,3,4] *

[1] Department of Occupational and Environmental Health, School of Public Health, Suzhou Medical College of Soochow University, Suzhou, China.

[2] Institute of Environmental Medicine, Karolinska Institutet, Box 210, 17177 Stockholm, Sweden.

[3] Faculty of Medicine, MV Lomonosov Moscow State University, 119991 Moscow, Russia

[4] Engelhardt Institute of Molecular Biology, RAS, 119991 Moscow, Russia

* Corresponding Author:

Boris Zhivotovsky: boris.zhivotovsky@ki.se

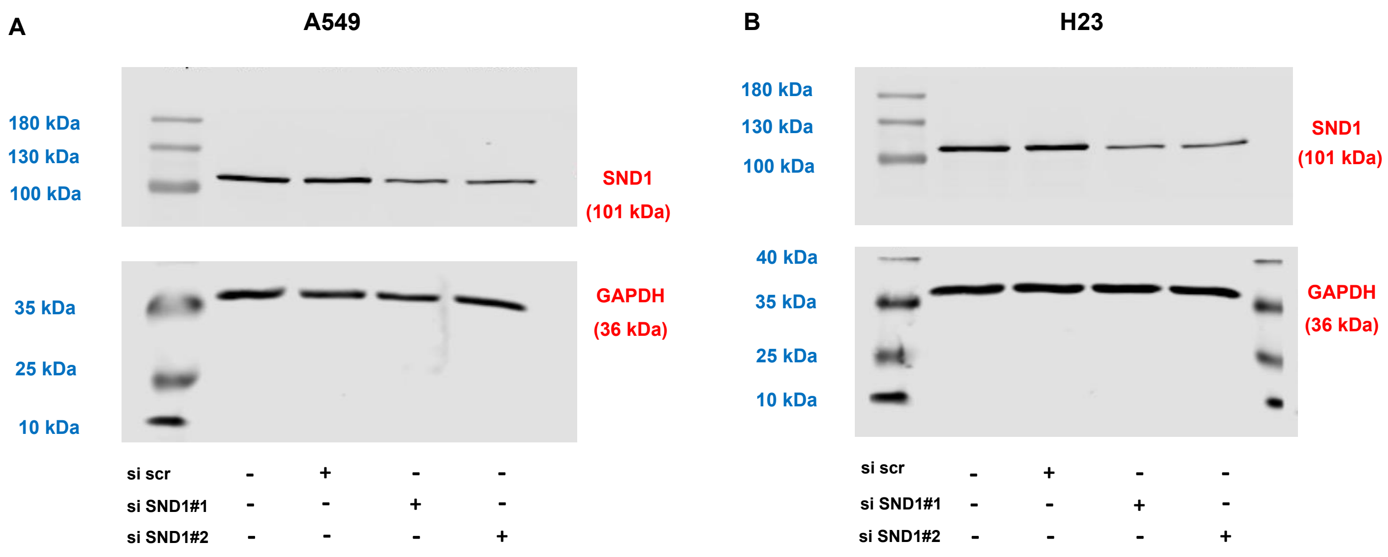

Figure SI1. Original images of western blotting are in Figure 1. A. Figure 1A_A549 cells. **B.** Figure 1C_H23 cells.

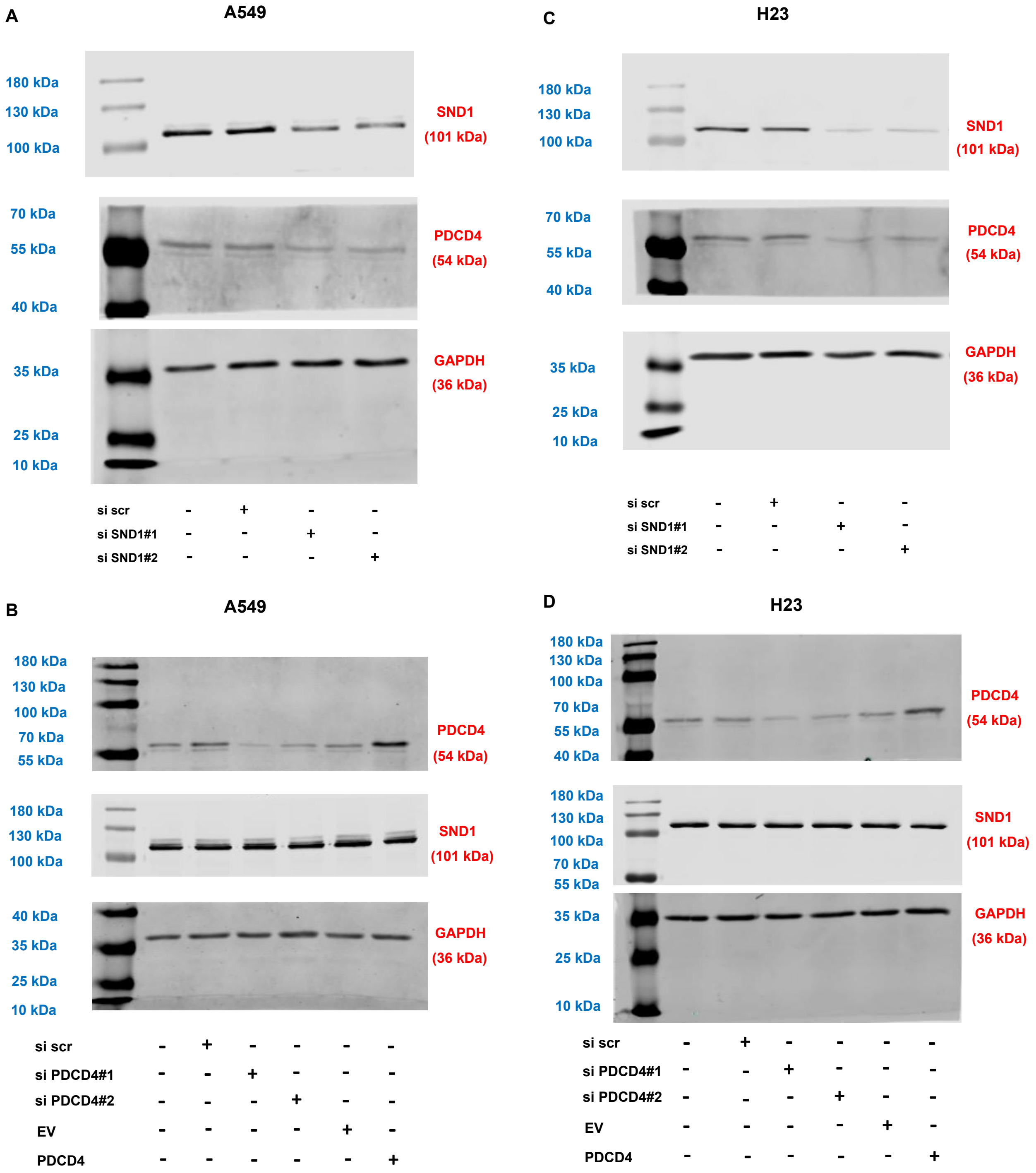

Figure SI2. Original images of western blotting in Figure 2. A. Figure 2A_A549 cells. **B.** Figure 2G_A549 cells. **C.** Figure 2D_H23 cells. **D.** Figure 2I_H23 cells.

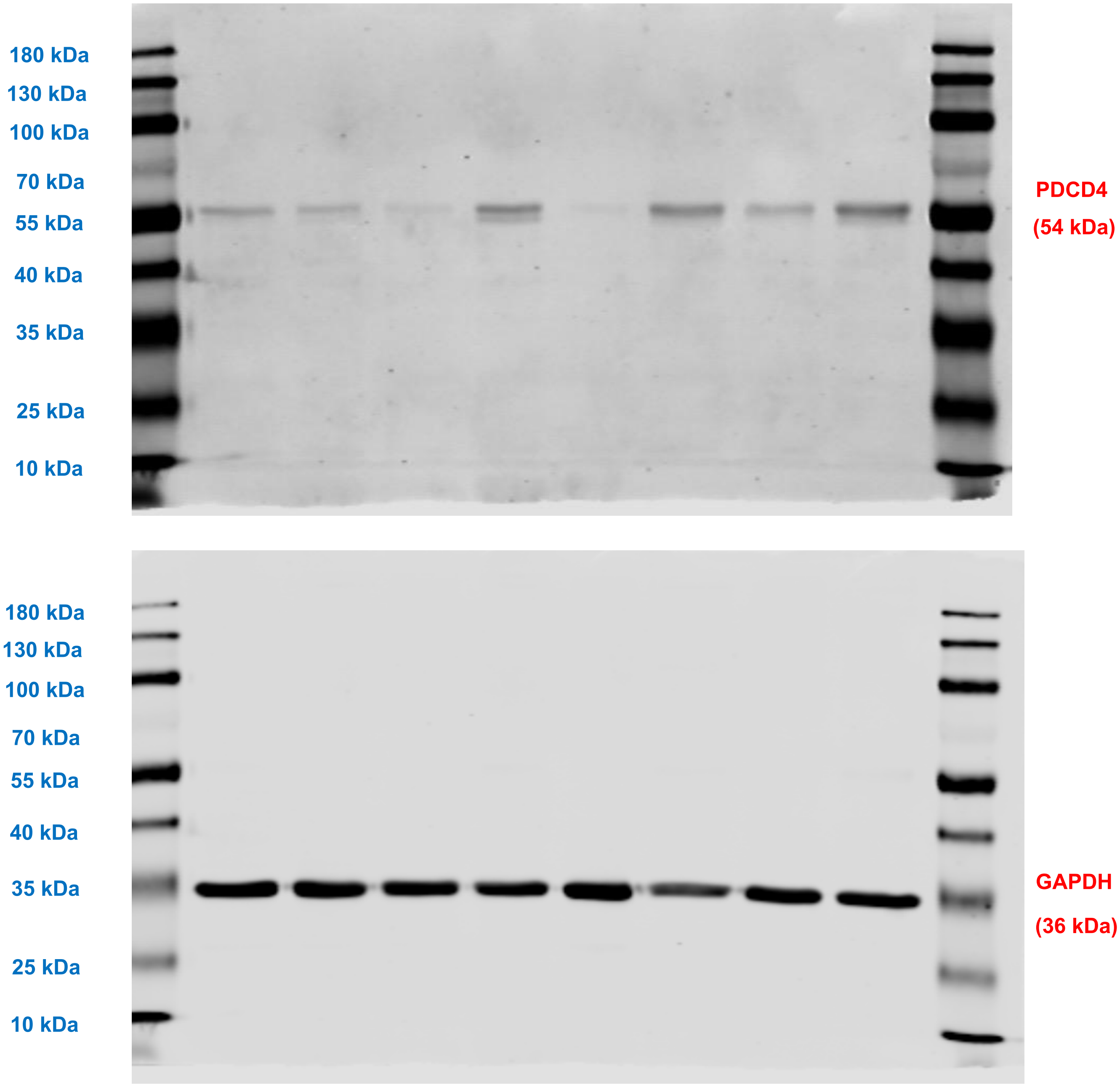

Figure SI3. Original images of western blotting in Figure 3B.

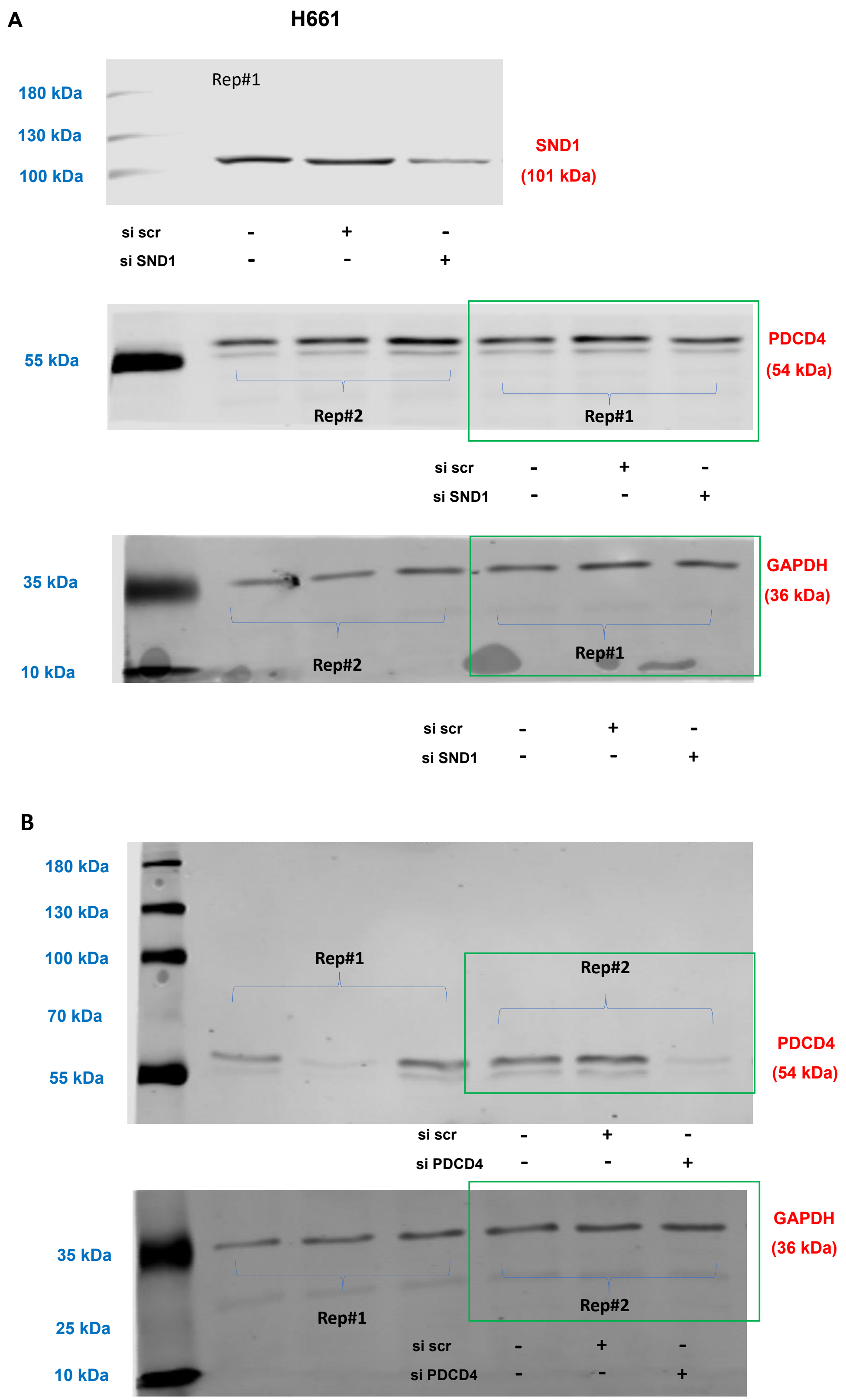

Figure SI4. Original images of western blotting in Figure 5. A. Figure 5A_H661 cells. **B.** Figure 5D_H661 cells.

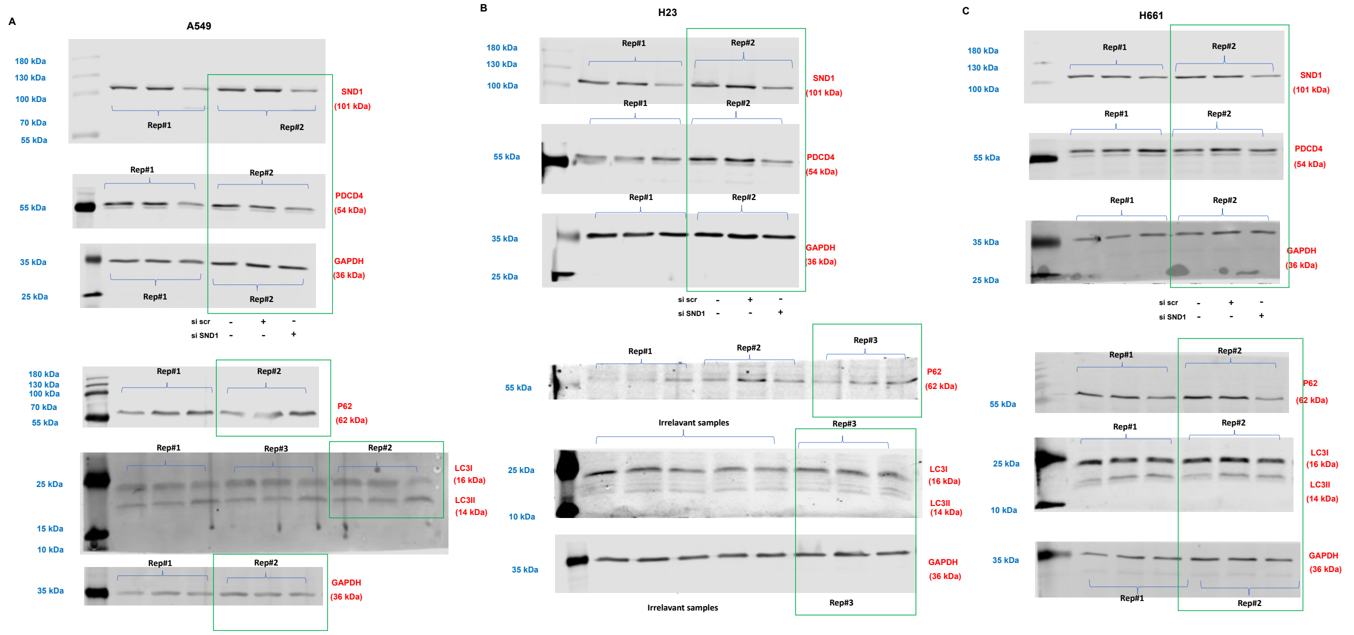

Figure SI5. Original images of western blotting in Figure 6. A. Figure 6A_A549 cells. **B.** Figure 6D_H23 cells. **C.** Figure 6G_H661 cells. The bands in the green boxes refer to the specific blotting bands showed in the manuscript.
